# Intra-operative radiological margins assessment in conservative treatment for non-palpable DCIS: correlation to pathological examination and re-excision rate

**DOI:** 10.1186/2193-1801-2-243

**Published:** 2013-05-24

**Authors:** Federico Buggi, Matteo Mingozzi, Annalisa Curcio, Camilla Rossi, Oriana Nanni, Lucia Bedei, Paola A Sanna, Salvatore Veltri, Secondo Folli

**Affiliations:** Breast Unit, Morgagni-Pierantoni Hospital, Forlì, Italy; Biostatistics and Clinical Trials Unit, IRCCS Istituto Scientifico Romagnolo per lo Studio e la Cura dei Tumori (I.R.S.T.), Meldola, FC Italy; Oncology Unit, Morgagni-Pierantoni Hospital, Forlì, Italy

## Abstract

What constitutes an adequate surgical margin in partial mastectomy is still controversial: intra-operative specimen radiogram is commonly used during partial mastectomy for nonpalpable lesions in order verify the adequacy of the resection but what margin is to be considered “adequate” is still debatable.

An intraoperative specimen mammogram was performed during all consecutive conservative resections for nonpalpable DCIS and a 15-mm radiological margin was considered “adequate”. Margins were pathologically assessed and classified as “negative”, “close” or “positive” and the rate of margin involvement constitued the main outcome of the study.

Among 272 conservative interventions, 80.51% had negative margins at final pathology, 3.31% had close margins and 16.18% had positive margins.

An intraoperative “adequate” margin of 15 mm as defined on intraoperative specimen mammogram granted a high rate of histologically negative margin at primary surgery; this finding was paralleled by confirmation of the treatment as conservative in 95% of cases.

**Electronic supplementary material:**

The online version of this article (doi:10.1186/2193-1801-2-243) contains supplementary material, which is available to authorized users.

## Introduction

Partial mastectomy is considered to be optimally performed by achieving adequate surgical margins during the initial operation while maintaining maximum cosmetic appearance of the breast (McCahill et al. 2012). When treating DCIS, such a perspective stems from either oncological concerns and quality assessment measures: from the oncological viewpoint, margins involvement by DCIS after breast-conservative treatment has been demonstrated to be associated with an approximate twofold overall increased risk of invasive ipsilateral breast tumor recurrence (Fisher et al. 1999;Wapnir et al. 2011) and, as far as quality outcomes are concerned, re-excisions increase the risk of postoperative infections, negatively impact cosmesis and increase medical costs due to a longer hospital stay (Thill et al. 2011); besides, re-excision may significantly alter a patient’s initial choice of treatment due to the impossibility of performing conservative operations as a secondary surgery in a high percentage of cases (10-36%) (McCahill et al. 2012).

Yet, what constitutes an adequate surgical margin in partial mastectomy is still an open question and, in facts, failure to achieve appropriate margins at the initial operation may lead to re-excision in up to 30-60% of cases (McCahill et al. 2012).

In patients undergoing localization lumpectomy for non-palpable breast cancer, it was suggested that a reliable peri-operative predictor of margin involvement could guide the extent of excision and therefore reduce the drawbacks that come along with re-operation (Reedijk et al. 2012).

Intra-operative specimen radiogram is widely used in partial mastectomy for non-palpable lesions in order to verify the adequacy of the resection but, to the best of our knowledge, reports on the actual assessment method are scanty and the ideal method for assessing the adequacy of excision remains elusive (Chagpar et al. 2003).

We report the criteria that were adopted for defining margins adequacy on intra-operative specimen mammogram in a series of conservative treatments for non-palpable DCIS and the status of the specimens margins as resulted at final pathology.

## Materials and methods

All consecutive patients conservatively operated from January 2001 to December 2009 due to preoperative stereotactic core biopsy positive for DCIS were included in the study. Patients diagnosed with DCIS who underwent primary mastectomy as well as those with palpable lesions were not included; patients treated with completion mastectomy as secondary surgery were included in the assessment of margin involvement after primary surgery but were excluded from the evaluation of ipsilateral breast tumor recurrence (IBTR).

Object of the study is a workflow outlined as follows: the biopsy site is marked with a clip at the time of the diagnostic procedure and the same clip is targeted with technetium-99 m-macroaggregate albumin the afternoon prior to surgery according to the ROLL technique (Gennari et al. 2000) (Radioguided Occult Lesion Localization); if the clip appears to be displaced, the target of the ROLL is the centre of the pathologic area. Multiple seeds are used to bracket large lesions or areas of microcalcifications at the radiologist’s discretion.

**Figure 1:**
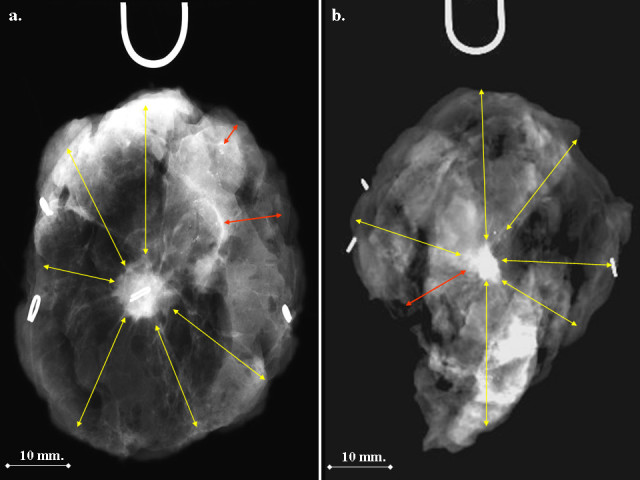
**Intraoperative specimen mammograms.** Yellow arrows: adequate (15 mm. or more) radiological margin. Red arrows: inadequate radiological margin from a cluster of microcalcifications (**a**) or tumor boundary (**b**).

During surgery, an intra-operative digital mammogram of the specimen is taken in order to verify the presence of the clip and the lesion as outlined on preoperative mammograms. Preoperative ROLL and intra-operative radiograph are routinely performed by the same radiologist and the correct orientation of the specimen is assured by the placement of stitches and radiopaque clips on the sample during surgery according to a written standard pattern, reported on a dedicated form. It presupposes the placement of two stitches on the cephalad margin (each one marked with a radiopaque surgical clip on its very base), one stitch on the caudal margin (marked with a single radiopaque surgical clip positioned at the base) and a different suture (with no radiopaque marker) on the margin close to the nipple; this margin is indicated during the mammogram by a U-shaped metal marker (Figure 1). If no skin is removed with the specimen, the superficial margin is inked. No compression is used; radio-transparent props are used if necessary to optimize the orientation of the specimen.

The distance from the boundary of the tumor (or from the outermost microcalcification) to the edge of the specimen is assessed in all directions on the digital mammogram and additional tissue is removed where it measures less then 15 mm (Figure 1).

In order to pursue a good cosmetic outcome, oncoplastic techniques are employed on demand, according to the criteria outlined by Clough et al. (2010).

Histopathological examination is conducted with tangential method (shaved, “en face”).

Pathology reports were reviewed and specimens margins classified as “negative” if the microscopic distance from the tumor was at least 2 mm, as “close” if comprised between 1.9 and 1 mm and as “positive” if lesser than 1 mm. With the purpose of statistical analysis, margin status was classified as “negative” versus “non-negative” (grouping “positive” and “close”).

Tumor grading was scored according to the Nottingham combined histological grading system (Elston & Ellis 1991). Immuno-histochemistry was used to assess ER- and PgR-status and Ki67. Fluorescence in-situ hybridization (FISH) was employed to determine HER2 amplification. ER- and PgR-status were considered “positive” if at least 10% of cells stained and otherwise “negative”. Ki-67 expression was classified as “high” if at least 20% of cells stained or “low”. HER2 amplification was defined according to guidelines issued by the American College of Pathologists (Lester et al. 2009).

The specimens of patients who underwent re-excision were analyzed with the same methods used for primary surgery; residual tumor was reported as “present” or “absent”.

Secondary surgery was indicated according to clinical judgement, using criteria similar to those reported by others (Morrow et al. 2012); indications were: age below 70, unfavorable biological features of tumor, extensive infiltration of a single margin or more than one margin infiltrated. On the other hand, indications to post-operative radiotherapy and adjuvant treatment (without re-operation) were: age above 70, favorable biological features of tumor, infiltration of one margin only.

The administration of radiotherapy was recorded and Patients were regularly followed-up after surgery. Adjuvant hormonal therapy is not routinely delivered for non-infiltrating carcinomas at our institution and occasional indications were issued upon individual-case multidisciplinary discussion.

Main outcome of the study was the rate of margin involvement at pathology. Secondary outcomes were re-excision rate and IBTR rate.

The database was anonymized assigning to each patient a unique identifier and deleting sensitive data. When anonymized administrative and clinical data are used to inform health care planning activities, the study is exempt from notification to the Ethics Committee and no specific written consent is needed to use patient information stored in the hospital databases.

An IBTR was defined as the occurrence of DCIS or invasive cancer in the treated breast. For each patient, time to failure was collected for all IBTRs, defined as time from the definitive surgery to the first occurrence of IBTR. Patients without IBTR were censored at the date of last follow-up. The date and type of recurrence (invasive vs. *in situ*), and the last date of follow-up were recorded for each patient after initial treatment.

The cumulative incidence of local relapse was estimated by Kaplan-Meier method (1958). The correlation between margin involvement and other clinico-pathological variables was assessed using a multiple logistic regression model in univariate and multivariate analyses.

All p-values were based on two-sided testing (p < 0.05) and statistical analysis was performed by using SAS statistical software (version 9.3, SAS Institute, Cary, NC). No formal sample size was planned, no multiplicity p value correction was implemented.

## Results

From January 2001 to December 2009, 339 patients underwent conservative surgery because of preoperative diagnosis of DCIS; 67 patients whose final pathology report showed coexistent infiltrating carcinoma (either at primary surgery or at re-operation) were excluded and therefore 272 patients constitute the study population.

At final pathology, 219 specimens (80.51%) had negative margins, 9 (3.31%) had close margins and 44 (16.18%) had positive margins (Table 1). Among the 53 non-negative margins, no further surgery was indicated in 25 (47.2%) cases while re-operation was undertaken in 28 (52.8%) cases (corresponding to 10.3% of the whole series), performing re-excision in 13 cases and completion mastectomy in 15; no residual tumor could be detected in the specimens from secondary surgery in 9 cases (32.1%).

**Table 1:** **Non-negative margins in 272 consecutive conservative surgical treatment**

	Number of involved margins			Total
	**one**	**two**	**three**	
“Positive” margins	30	12	2	44
“Close” margins	8	1	-	9
Total	38	13	2	53

Median patients age was 58 (range:30–83). Nuclear grading was scored G1 in 47 (17.9%) cases, G2 in 115 (43.9%) and G3 in 100 (38.2%); it was not assessed in 10 patients. Tumors proved ER-positive in 122 cases (74.4%) and ER-negative in 42 (25.6%); PgR resulted positive in 101(61.6%) cases and negative in 63 (38.4%). Hormone receptors were not determined in 108 cases. Ki-67 expression was “high” in 20 cases (12.5%) and “low” in 140 cases (87.5%); it was not assessed in 112 cases. HER2-status was tested in 53 cases and resulted amplified in 16 cases (30.2%) (Table 2).

**Table 2:** **Biological features of 272 DCIS**

	n. (%)		Non-negative	Negative
			Margins^a^	Margins
			(N = 53)	(N = 219)
Age				
24 ≤50	81	(29.7)	16	65
>50	191	(70.3)	37	154
ER status				
Negative	42	(25.6)	6	36
Positive	122	(74.4)	30	92
Unknown	108			
PgR status				
Negative	63	(38.4)	13	50
Positive	101	(61.6)	22	79
Unknown	108			
Ki-67 proliferative index				
<20%	140	(87.5)	31	109
≥20%	20	(12.5)	5	15
Unknown	112			
HER2 status				
Amplified	16	(30.2)	9	7
Not amplified	37	(69.8)	8	29
Unknown	219			
Grading				
G1	47	(17.8)	2	45
G2	115	(43.9)	22	93
G3	100	(38.2)	23	77
Unknown	10			

*ER*: Estrogen Receptor.

*PgR*: Progesterone Receptor.

*HER2*: Human Epidermal growth factor Receptor 2.

^a^ “Non-negative” margins include margins classified either as “positive” or as “close” (see text).

As stated above, 15 patients with non-negative margins after primary surgery who underwent completion mastectomy were excluded from further analysis of recurrence rate; therefore, 257 cases were candidate to breast conservative treatment: 207 (81.5%) received post-operative radiotherapy while 47 (18.5%) did not (in 3 cases data were not available).

After a median follow-up of 63 months (range: 7–126), 22 IBTRs were observed (8.56%), as DCIS in 10 cases (45%) and as infiltrating carcinomas in 12 (55%); 17 recurrences took place in patients whose specimen margins had resulted “negative” while in 5 cases the margins had resulted “close” (1 case) or “positive” (4 cases).

**Figure 2:**
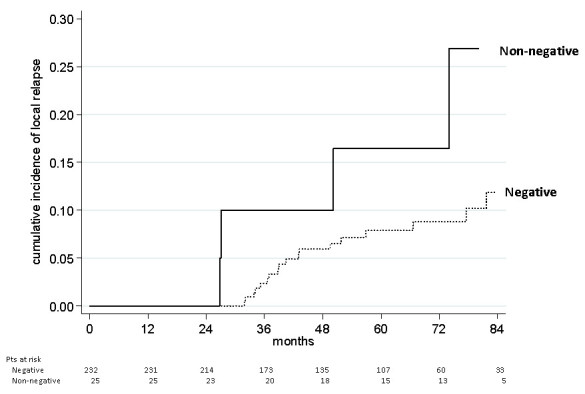
**Cumulative incidence of local relapse according to margin status.**

Overall, 5-year relapse cumulative incidence was 9.2% (95% CI: 5.9-14.3). Five-year relapse cumulative incidence was 8.5% (95% CI: 5.2-13.8) for negative margins and 16.4% (95% CI: 5.5-43.2) for positive margins (Figure 2).

## Discussion

Breast cancer treatment requires a multidisciplinary approach and it’s recommendable that an organized pathway through the disciplines involved is granted to patients in order to optimize clinical outcomes. The purpose in the treatment of DCIS is to maximize local control with the least-aggressive treatment (Kumar & Sacchini 2010). The extent of disease within the breast and the presence of multifocality, which in turn affect the ability to achieve negative margins, heavily influence surgical planning (Dick et al. 2011) but, unfortunately, precise preoperative assessment of DCIS extension remains elusive because traditional clinical evaluation proved unreliable (Holland et al. 1985) and recent diagnostic tools showed some limitations as well. MRI can in facts detect DCIS (in particular high- or intermediate-grade lesions) but it does not accurately predict the size of the tumor and does not improve the surgeon's ability to achieve clear margins following breast-conservative surgery (Kropcho et al. 2012).

As far as intraoperative evaluation is concerned, Chagpar et al. (2003) claimed that radiologic assessment alone is insufficient for accurate evaluation of margin status, because margins that appear negative on specimen radiography may be histologically positive in up to 44% of cases. Therefore, a technique for intraoperative margin assessment that includes sliced specimen radiography and gross pathological examination was reported; the addition of gross pathological examination led to a 28%-rate of intraoperative negative margins that actually resulted positive on final pathology; these results appear slightly worse than the 19.5% rate of positive margins at final pathology that we observed. Unfortunately, as reported by the Chagpar et al., frozen section analysis is not felt to be indicated in cases with microcalcifications because it may induce artifacts and leave insufficient unfrozen tissue for permanent sections, so in our opinion the radiological margin that we adopted allowed to overcome some limitations existing in intraoperative pathological examination.

In the present series, a consistent radiological margin was used and eventually 219 cases (80.51%) out of 272 conservative treatments had negative margins at pathology after primary surgery; among the 53 non-negative margins, the majority (71.7%) had only one margin involved, either as “positive” or “close”. Re-operation was undertaken in 28 cases (10.3%), requiring completion mastectomy in 15 cases (5.5%); therefore, the treatment of 257 cases out of 272 (94.5%) was eventually confirmed as conservative.

The re-operation rate that we recorded (10.3%) compares favorably, for instance, with the 60.5% reported by Rudloff in their observational study of 304 women with DCIS treated with breast-conserving therapy (Rudloff et al. 2010). In another population-based study (Morrow et al. 2009), a 42.7% rate of additional surgery is reported, either as re-excision lumpectomy (30.7%) or as post-BCS mastectomy (12%)*.*

However, re-excision rates reported in the literature are quite variable and the variation parallels different definitions of “adequate” margin, often not supported by consistent high-quality clinical data (Azu et al. 2010).

Considerable debate also concerns the definition of “negative” margin and whether the width of a negative margin (width of a margin negative for tumor cells) is associated with a decreased risk of recurrence. Some evidence in the literature supports the conclusion that wider negative margins confer the greatest protection (Kerlikowske et al. 2003), but no study comparing women whose margins were above or below a specific threshold found any benefit to being above vs. below the threshold (Virnig et al. 2010). Besides, a significant proportion of re-excisions are reported to be done in patients with negative margins (Azu et al. 2010).

Due to uncertainty about “adequate”margin, indication to secondary surgery is to some extent subjective and our work constitutes no exception, but it is worth noticing that in the present series no residual tumor in the specimens from secondary surgery could be detected in only 9 cases (32.1%), meaning that the choice of no surgical indication in non-negative margin cases would have left in place residual cancer in 67.9% of cases. Within the frame of a low margin-involvement rate, we consider this figure an estimate of consistent predictive value of the margins assessment we adopted (and a proxy of sound indications to secondary surgery).

Within a low overall reoperation rate, in our series a mastectomy was undertaken roughly half of the times that a secondary surgery was indicated, and therefore indications to BCT were eventually turned into a demolitive operation in 5.5% of cases. This figure may be interpreted as a fulfillment of patients’ cosmetic demands, but in the present study no survey of patient satisfaction concerning cosmetic outcome was carried on and this may be considered as a limit of the work.

After a median follow-up of 63 months we recorded a 8.56% relapse rate, which compares similarly with the Silverstein’s series (1999), where an IBTR rate as low as 6% at 5 years was reported for patients with small lesions, favorable histologies, and low/intermediate grade with widely negative margins (>1 cm) treated by BCS alone. Our results may be influenced by the low proportion of high grade DCIS (38.2%), the presence of mostly ER-positive (74.4%) lesions and the high proportion of patients receiving post-operative radiotherapy (80.5%). In facts, there are conflicting retrospective data demonstrating higher local relapse rates even in these favorable patients groups, with the omission of radiation therapy (Motwani et al. 2011). As reported in the series by Rudloff (2010), in the most favorable subgroup (margin width ≥10 mm, older age, absent palpable mass, absent lobular neoplasia), the 10-year cumulative IBTR rate was 13% without RT. Unfortunately, the small numbers in our series did not allow a comparison between patients who underwent RT and those who did not. Besides, long follow-up periods appear to be necessary in order to be able to detect IBTRs. In the randomized National Surgical Adjuvant Breast Project (NSABP) B-17 trial and in the European Organization for Research and Treatment of Cancer (EORTC) trial, the rates of IBTR increased by nearly 40% after 5 years (Motwani et al. 2011); in the overall series reported by Rudloff (2010), 21% of recurrences took place after a median follow-up of 11 years and actuarial 10- and 15-year IBTR rates for all patients were 22% and 29%, respectively. In the series by Motwani (2011) the majority of local relapses occurred after 5 years and this late development was more apparent in the low/intermediate grading cohort (75%) versus the high grade cohort (50%). Therefore IBTR rates will likely increase in our series, as the follow-up period extends.

In conclusion, in our experience an intra-operative “adequate” margin of at least 15 mm as defined on intraoperative mammogram of the specimen has allowed to obtain a high rate of histologically negative margin at primary surgery (80.51%); this finding was paralleled by a 10.3% reoperation rate, with a high (94.5%) likelihood of eventually maintain indications to conservative surgery, often permitted by recourse to oncoplastic techniques. Hopefully, further studies will allow to understand whether smaller radiological margins grant low margin positivity on final pathology after primary surgery.

## Authors’ original submitted files for images

Below are the links to the authors’ original submitted files for images. Authors’ original file for figure 1 Authors’ original file for figure 2

## References

[CR1] Azu M, Abrahamse P, Katz SJ, Jagsi R, Morrow M (2010). What is an adequate margin for breast -conserving surgery? surgeon attitudes and correlates. Ann Surg Oncol.

[CR2] Chagpar A, Yen T, Sahin A (2003). Intraoperative margin assessment reduces reexcision rates in patients with ductal carcinoma *in situ* treated with breast-conserving surgery. Am J Surg.

[CR3] Clough KB, Kaufman GJ, Nos C, Buccimazza I, Sarfati IM (2010). Improving breast cancer surgery: a classification and quadrant per quadrant atlas for oncoplastic surgery. Ann Surg Oncol.

[CR4] Dick AW, Sorbero MS, Ahrendt GM (2011). Comparative effectiveness of ductal carcinoma *In situ* management and the roles of margins and surgeons. J Natl Cancer Inst.

[CR5] Elston CW, Ellis IO (1991). Pathological prognostic factors in breast cancer I. The value of histological grade in breast cancer: experience from a large study with long-term follow-up. Histopathology.

[CR6] Fisher B, Dignam J, Wolmark N (1999). Tamoxifen in treatment of intraductal breast cancer: national surgical adjuvant breast and bowel project B-24 randomised controlled trial. Lancet.

[CR7] Gennari R, Galimberti V, De Cicco C (2000). Use of technetium-99m-labeled colloid albumin for preoperative and intraoperative localization of nonpalpable breast lesions. J Am Coll Surg.

[CR8] Holland R, Velig SHJ, Mravunac M, Hendriks JHCL (1985). Histologic multifocality of Tis, T 1–2 breast carcinomas. Cancer.

[CR9] Kaplan EL, Meier P (1958). Non parametric estimation from incomplete observation. J Am Stat Assoc.

[CR10] Kerlikowske K, Molinaro A, Cha I (2003). Characteristics associated with recurrence among women with ductal carcinoma *In situ* treated by lumpectomy. J Natl Cancer Inst.

[CR11] Kropcho LC, Steen ST, Chung AP, Sim MS, Kirsch DL, Giuliano AE (2012). Breast MRI in the surgical treatment of ductal carcinoma *in situ*. Breast J.

[CR12] Kumar S, Sacchini V (2010). The surgical management of ductal carcinoma *in situ*. Breast J.

[CR13] Lester SC, Bose S, Chen YY (2009). Protocol for the examination of specimens from patients with invasive carcinoma of the breast. Arch Pathol Lab Med.

[CR14] McCahill LE, Single RM, Aiello Bowles EJ (2012). Variability in reexcision following breast conservation surgery. JAMA.

[CR15] Morrow M, Jagsi R, Alderman AK (2009). Surgeon recommendations and receipt of mastectomy for treatment of breast cancer. JAMA.

[CR16] Morrow M, Harris JR, Schnitt SJ (2012). Surgical margins in lumpectomy for breast cancer -bigger is not better. N Engl J Med.

[CR17] Motwani SB, Goyal S, Moran MS, Chhabra A, Haffty BG (2011). Ductal carcinoma *in situ* treated with breast-conserving surgery and radiotherapy: a comparison with ECOG study 5194. Cancer.

[CR18] Reedijk M, Hodgson N, Gohla G (2012). A prospective study of tumor and technical factors associated with positive margins in breast-conservation therapy for nonpalpable malignancy. Am J Surg.

[CR19] Rudloff U, Brogi E, Reiner AS (2010). The influence of margin width and volume of disease near margin on benefit of radiation therapy for women with DCIS treated with breast-conserving therapy. Ann Surg.

[CR20] Silverstein MJ, Lagios MD, Groshen S (1999). The influence of margin width on local control of ductal carcinoma *in situ* of the breast. N Engl J Med.

[CR21] Thill M, Röder K, Diedrich K, Dittmer C (2011). Intraoperative assessment of surgical margins during breast conserving surgery of ductal carcinoma *in situ* by use of radiofrequency spectroscopy. The Breast.

[CR22] Virnig BA, Tuttle TM, Shamliyan T, Kane RL (2010). Ductal carcinoma *in situ* of the breast: a systematic review of incidence, treatment, and outcomes. J Natl Cancer Inst.

[CR23] Wapnir IL, Dignam JJ, Fisher B (2011). Long-term outcomes of invasive ipsilateral breast tumor recurrences after lumpectomy in NSABP B-17 and B-24 randomized clinical trials for DCIS. J Natl Cancer Inst.

