# Supplementary figures and images for: Intra-operative radiological margins assessment in conservative treatment for non-palpable DCIS: correlation to pathological examination and re-excision rate
Source: Springerplus. 2013 May 24;2(1):243. doi: 10.1186/2193-1801-2-243 (PMC3669500; doi:10.1186/2193-1801-2-243)

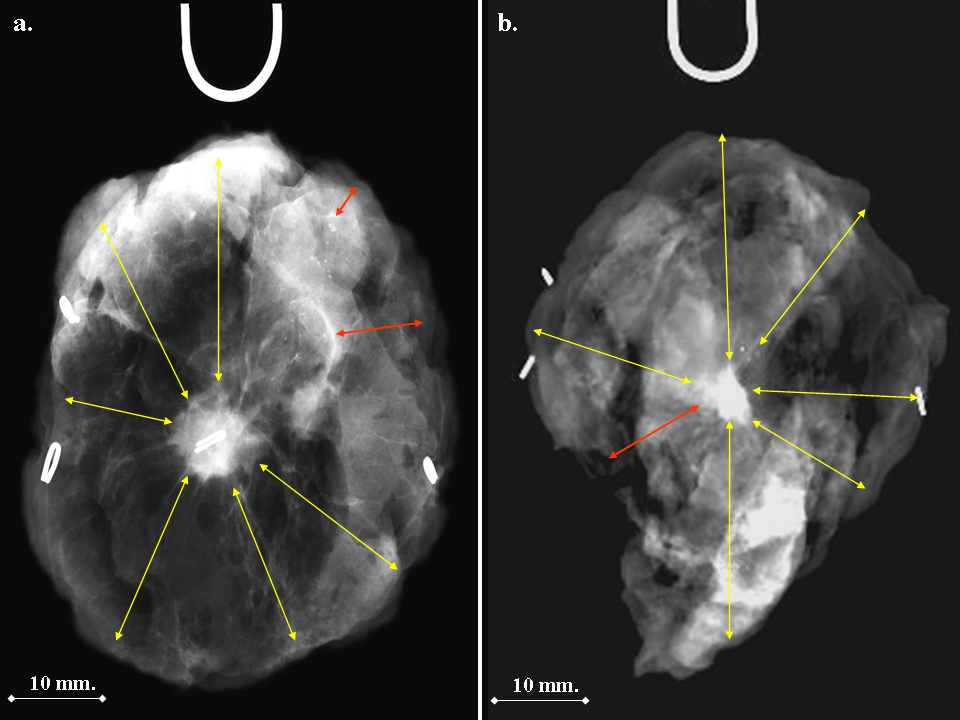

Supplement: Supplementary file 1 — Authors’ original file for figure 1 [file 40064_2013_300_MOESM1_ESM.tiff]

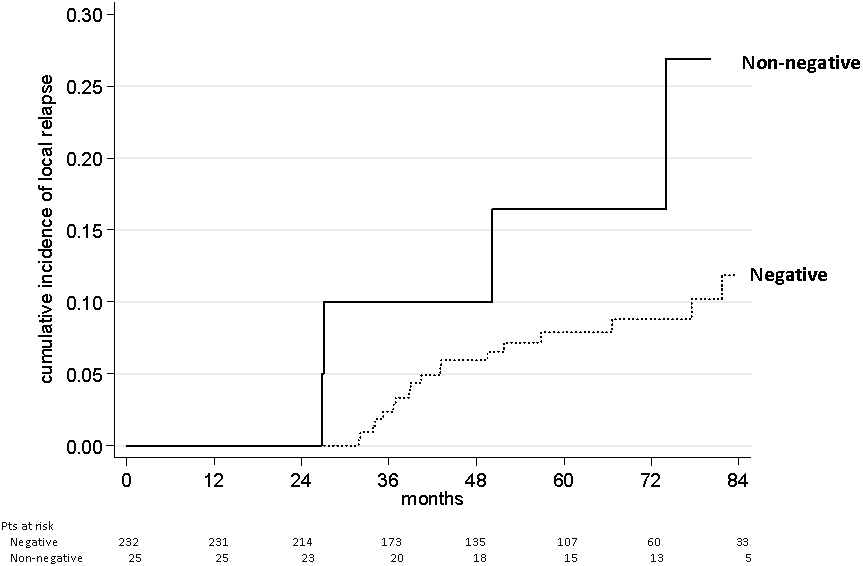

Supplement: Supplementary file 2 — Authors’ original file for figure 2 [file 40064_2013_300_MOESM2_ESM.tiff]
